# Evaluation of Hospitalizations for Tick-Borne Diseases in the United States from 2002 to 2021

**DOI:** 10.3390/tropicalmed10090238

**Published:** 2025-08-27

**Authors:** Sidhvi Nekkanti, Kirsten Hickok, Mahesh Shrestha, Eric Edewaard, Thomas A. Melgar

**Affiliations:** 1 Western Michigan Homer Stryker M.D. School of Medicine, Kalamazoo, MI 49009, USA; 2 Department of Biomedical Informatics, Western Michigan Homer Stryker M.D. School of Medicine, Kalamazoo, MI 49009, USA; kirsten.hickok@wmed.edu; 3 Department of Pediatrics and Adolescent Medicine, Western Michigan Homer Stryker M.D. School of Medicine, Kalamazoo, MI 49009, USA; mahesh.shrestha@wmed.edu; 4 Department of Internal Medicine, Western Michigan Homer Stryker M.D. School of Medicine, Kalamazoo, MI 49009, USA; eric.edewaard@wmed.edu; 5 Departments of Pediatrics, Adolescent and Internal Medicine, Western Michigan Homer Stryker M.D. School of Medicine, Kalamazoo, MI 49009, USA; thomas.melgar@wmed.edu

**Keywords:** tick-borne diseases, lyme disease, babesiosis, anaplasmosis, ehrlichiosis, rocky mountain spotted fever, epidemiology, hospitalization

## Abstract

Tick-borne diseases (TBDs) are a growing public health concern in the United States. This study analyzed 261,630 weighted hospitalizations from the Healthcare Cost and Utilization Project (HCUP) Nationwide Inpatient Sample (NIS) database between 2002 and 2021 to evaluate trends, coinfections, demographic disparities, and financial impacts. Lyme disease was the most common cause, accounting for 65% of hospitalizations (171,328 admissions), followed by ehrlichiosis/anaplasmosis (46,446), babesiosis (18,057), rickettsial diseases (16,412), tularemia (2428), and other TBDs (19,435). Hospitalizations increased 2.5-fold over the study period, with the Northeast region bearing 52.9% of the burden and peaking in July. Males (53.9%), Caucasians (81.4%), and residents of higher-income zip codes were predominant, though rickettsial diseases showed elevated Hispanic representation (18.4%). Coinfections were common, with 35.8% of babesiosis and 15.6% of ehrlichiosis/anaplasmosis cases involving another TBD, suggesting that routine testing may be warranted. Median hospital charges rose from USD 9433 in 2002 to USD 35,161 in 2021, totaling USD 1.265 billion in 2021. In-hospital mortality was 1.1%, with the highest cause of mortality being babesiosis (2.06%). Future areas for research include characterizing the burden of disease in an outpatient setting, understanding the causes of racial disparities in hospitalizations, and testing strategies to identify coinfection.

## 1. Introduction

Tick-borne diseases (TBDs) are a significant public health concern globally, but also in the United States (US) [1]. Among these, Lyme disease, caused by *Borrelia burgdorferi* and transmitted primarily by *Ixodes scapularis* ticks in the northeastern and upper Midwest U.S., is the most prevalent [2]. However, there are many TBDs in the United States that contribute to morbidity and mortality [3,4]. The true global burden of TBDs is likely underestimated. There is concern about underreporting, misdiagnosis, and limited diagnostic sensitivity. Studies suggest that Lyme disease cases may be underestimated by as much 12–15-fold [5].

Climate change is also expected to have a significant impact on the prevalence and geographic distribution of TBDs [6]. Increasing temperatures, milder winters, and changes in precipitation have all expanded regions of the United States that can be inhabited by tick vectors and their animal hosts. This expansion is currently seen in the Northeast and Upper Midwest, where reforestation and suburban changes to wooded areas have increased human exposure to tick habitats [7,8]. For example, the white-tailed deer is a host for the *Ixodes scapularis* and has seen a drastic rise in population following centuries of deforestation, adding to the increased risk of tick population expansion [9].

Historically, estimates of TBD burden have relied on reporting to public health entities. This method does not provide much understanding of the impact that these conditions have on the healthcare system and does not discriminate between mild and severe TBD cases. Hospitalization data offers the ability to quantify the burden of severe cases and delineate geographic and seasonal trends related to cases that are severe enough to require an inpatient stay [10]. Moreover, outpatient reporting is dependent on socioeconomic differences in access to care; therefore, severe cases requiring admission likely bypass this constraint. This highlights the need for robust data sources, like inpatient records, to better understand the impact of TBDs on healthcare systems and public health initiatives.

Therefore, this study utilizes a nationally represented inpatient database to characterize trends in hospitalizations for TBDs in the United States. This database identifies diagnoses using ICD codes, which are standardized alphanumeric codes used to classify and record diagnoses during healthcare encounters. The insights from this study are crucial for informing public health surveillance initiatives, bringing awareness in the clinical environment, and providing strategies to mitigate the impact of these diseases.

## 2. Methods

This research utilized a retrospective observational study design analyzing data from the Healthcare Utilization Project (HCUP) nationwide inpatient sample (NIS) database, covering hospitalizations across various regions in the United States between 2002 and 2021. The NIS database is a publicly available de-identified database and contains a sample of 20% of all inpatient admissions in the United States. The data is systematically collected at the time of discharge to allow clustering and weighting in order to extrapolate results to represent 100% of the hospital admissions during the calendar year and the total of all hospital admissions in the United States can be reliably reported. The database contains data from approximately 7 million hospital admissions per year, representing approximately 35 million weighted admissions each year, or more than 700 million admissions during the study time period.

Data variables include the total number of admissions each year, demographics such as age, sex, race/ethnicity, payor, hospital type (academic vs. non-academic), and setting (rural vs. urban). Average household income within the zip code of the patient’s residence is used as a surrogate for the patients’ actual household income status. In-hospital mortality data is available; however, data on the rates of mortality after discharge is not available. Length of stay and total hospital charges are included. All billing and procedure codes for each hospitalization (up to 100 codes) are recorded. Codes are prioritized by the attending physicians as well as hospital coders, ranked based on their importance and relevance to the hospital admission.

Data was analyzed by the Western Michigan University Homer Stryker, MD School of Medicine biostatistics department, which has extensive experience working with this database. The data was managed securely in accordance with the WMed institutional policies, HIPAA regulations, and HCUP requirements. The data was rendered anonymous before analysis, with only the necessary information being retained for research purposes. All data was stored in encrypted databases, with access restricted to authorized research personnel. Data analysis included descriptive statistics to examine hospitalization trends, with regression models employed to assess socio-demographic and geographic factors associated with severe outcomes. Per NIS requirements, cells containing fewer than ten patients cannot be reported to the researchers or published due to the potential risk of re-identifying patients. These cells are noted with an *. Cells with zero cases can be reported.

Inclusion criteria (Table 1) were any admissions that had a diagnostic code for at least one of the tick-borne diseases (Lyme disease, babesiosis, anaplasmosis, ehrlichiosis, rickettsial diseases and tularemia and other TBDs) in one of the first two diagnoses at the time of discharge. ICD9 and ICD10 diagnostic codes for tick-borne diseases were categorized and listed. Data from the year 2015 were excluded due to changes in coding from ICD9 to ICD10 in October of that year, as well as incomplete data. Anaplasmosis was previously known as granulocytic ehrlichiosis and coded as ehrlichiosis in ICD9 prior to 2015. The addition of anaplasmosis as a diagnostic code in ICD10 required combining anaplasmosis with ehrlichiosis into one category since both were likely coded as ehrlichiosis prior to 2015. Additionally, changes in codes in the less-common TBDs along with the lack of specificity of some codes in ICD9 prevented the detailed analysis of those diseases. As a result, a single category of “other TBDs” was created.

Descriptive analysis was used to assess the frequencies of total and individual tick-borne diseases. The frequencies and proportions (95% confidence intervals [CI]) are reported for the study period. Categorical patient characteristics were reported as frequencies and percentages, while continuous patient variables are reported as medians and interquartile ranges. Data on four geographic regions—as defined by the HCUP protocol (Northeast, Midwest, South and West)—of the country was provided. More-detailed individual state-level data is not available.

Trends over time were plotted. The lengths of each season of admissions (recorded in months) for tick-borne diseases were analyzed using trends in standard deviations for each year, with more than 1000 hospital admissions identified for TBDs. A non-Poisson linear regression line was used to identify the best fit trend line. Weighted frequencies were reported, and all analyses were completed using weighted estimates in accordance with the NIS sampling methodology. SAS Studio software version 9.4 was used for the analysis.

## 3. Results

**Figure 1:**
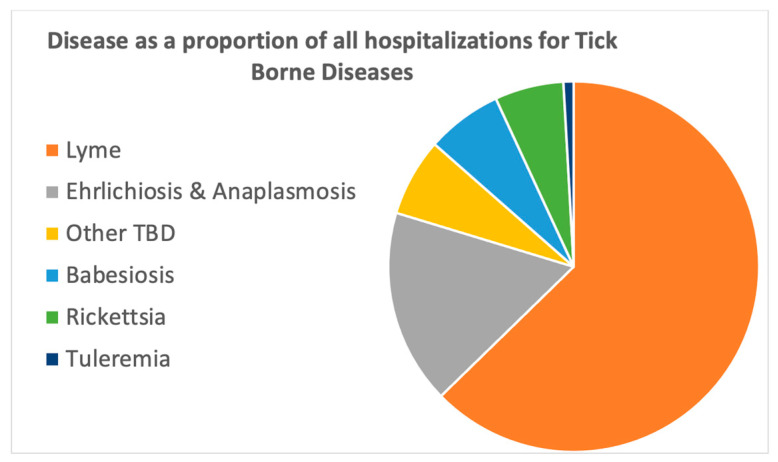
Relative proportion of TBD admissions by disease.

There was a total of 261,630 weighted hospital admissions for TBDs during the study period (Table 2). Lyme disease made up almost 2/3 (65%) of this total, with 171,328 hospital admissions (Table 3). Ehrlichiosis/Anaplasmosis was next most common, followed by babesiosis, rickettsia and tularemia (Figure 1). The “other TBD” group had 19,435 admissions and was the third-largest group; however, the individual diseases in this group had fewer admissions, and there was evidence that many of the diseases in this group were miscoded as other diseases within this same group.

**Figure 2:**
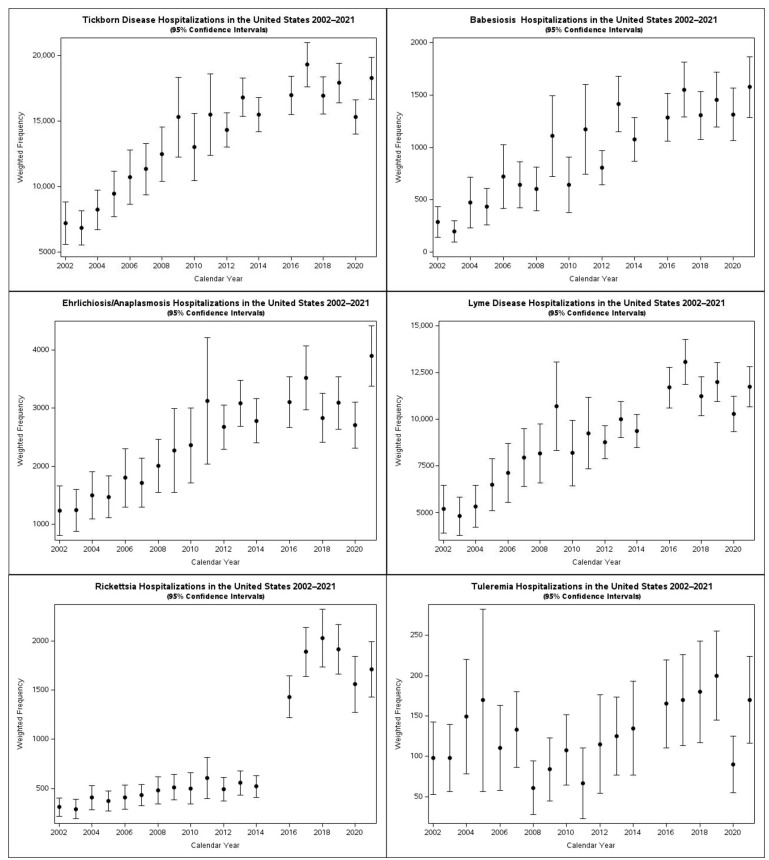
Weighted frequencies of tick-borne diseases by calendar year.

Frequency over time: During the study period, the total number of TBDs increased when pooled, as well as for each of the individual tick-borne diseases, although the confidence intervals for trends for tularemia are too wide to demonstrate a trend (Figure 2). The total number of admissions for TBDs increased in frequency by a factor of 2.5 over the 20-year study period. The trends for Lyme, babesiosis, and ehrlichiosis/anaplasmosis have been relatively stable over the last five years of the study period.

Gender and TBD—Males were affected more often than females—53.9% vs. 46.1%. (Table 2) These differences varied across all tick-borne diseases. Lyme disease had a slight female predominance of less than 1% difference, while hospital admissions for the others ranged from 59.9% to 66.3% male.

Race: Caucasians are over-represented in recorded numbers of hospitalizations overall (87%). It is important to note that the US Census number and NIS number for the Caucasian population is around 65%. Black and Hispanic patients showed a reciprocal under-representation for all TBDs, except for rickettsia. This disease showed an increase in cases in Hispanic patients (19.5%), which is consistent with the US census population data, but higher than the overall NIS racial distribution (11.5%).

Income: There were significant differences in frequencies of TBD hospitalizations among income levels when examined by home zip codes. Overall, patients from wealthier zip codes were more likely to be hospitalized with TBDs, with the highest-quartile income level having more than twice the number of TBDs compared with the lowest-quartile income level. This was not a consistent finding for all TBDs (Table 4). The impact of income among all TBDs pooled together was strongly influenced by an even greater difference with Lyme disease, the most frequent TBD admitted to the hospital, where the highest income quartile had nearly four times the frequency of Lyme disease hospitalization compared with the lowest income quartile. Babesiosis, while less common than Lyme disease, had an even greater difference, with the highest income quartile having ten times the frequency of the lowest income quartile; moreover, ehrlichiosis and anaplasmosis were twice as common in the highest vs. lowest income quartiles. Interestingly, for tularemia, rickettsial diseases, and other TBD hospitalizations, the opposite pattern was true, showing a negative association with income. Hospitalizations due to these diseases were two to three times more common in the lowest income quartile compared to the highest. For these diseases, the trends were linear across income groups.

**Figure 3:**
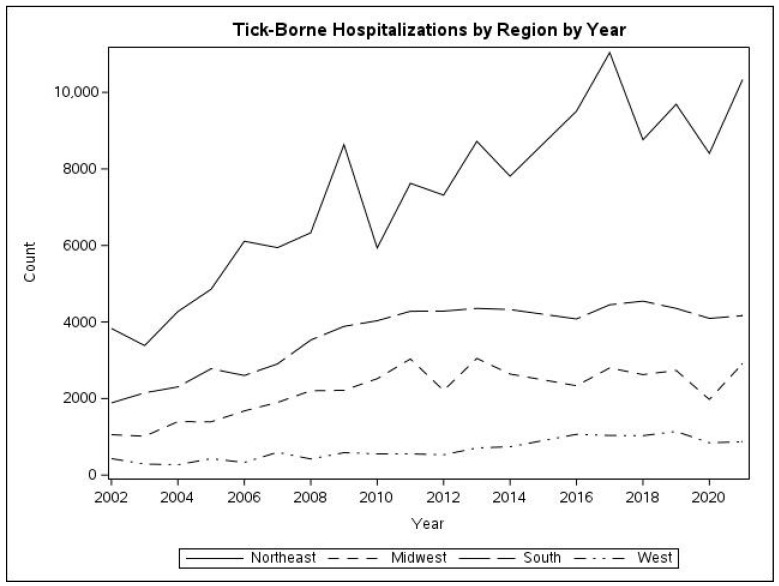
Change in TBD hospitalizations over time, across geographic regions of the United States.

Geographic distribution: Analysis of the geographic distribution of TBD admissions shows that more than half of all hospitalizations for TBDs occur in the Northeast. This was also strongly influenced by Lyme disease and babesiosis, with 60.1% and 86.9%, respectively, of hospitalizations for these diseases occurring in the Northeast. Ehrlichiosis and anaplasmosis were also more common in this area, while tularemia, rickettsia, and other TBDs were most common in the South. Figure 3 shows the temporal changes in frequency of all TBDs. There are similar increases in TBDs in all regions relative to their 2002 frequency.

Taken together, the income and geographic data suggests that in the Northeast, TBDs were positively correlated with income, while in the South, TBDs were negatively correlated with income. The data on income in each of the regions was not directly analyzed in this study.

Setting: Nearly 5 times as many hospitalizations for TBDs occurred in urban areas compared to rural areas. This pattern was true for all TBDs. The database does not include the term ‘suburban’. ‘Suburban’ is included in the urban category.

The financial burden of hospitalizations for TBDs: The financial burden of TBD hospitalization was analyzed. The median charges per hospitalization involving tick-borne diseases rose from USD 9433 to USD 35,161 (Table 5). The total charges for all admissions involving TBDs rose from USD 114 million in 2002 to USD 1.27 billion in 2021.

Co-infections: Analysis of the data on co-infections showed that concurrent infections with two or more TBD organisms are relatively common during hospitalizations with any TBD. Table 6 and Table 7. Rates varied depending on the disease considered. Overall, 5.5% of patients with Lyme disease had a co-infection with another TBD. Babesiosis had the highest co-infection rate—35.8% of patients admitted with babesiosis had at least one other TBD. Anaplasmosis/ehrlichiosis had a co-infection rate of 15.6%. Although co-infections with both anaplasmosis and ehrlichiosis are likely, due to coding issues in ICD9, we were unable to identify the co-infections between these two organisms. The frequency of co-infections increased throughout the study period.

Mortality: The overall mortality for all hospitalizations with a TBD was low, at 1.1%. This was also relatively consistent across all types of TBD, with babesiosis having the highest rate at 2.06%. With babesiosis having the highest frequency of co-infection and the highest mortality, the possibility that co-infections had a higher rate of mortality was investigated. The odds ratio for mortality in patients with two or more concurrent TBD infections relative to those with a single infection was not significant [1.18 (95% CI 0.76, 1.83)]. Males had a slightly higher mortality rate compared with females [1.20 (95% CI 1.01, 1.42)]. There were no significant differences in mortality between household income quartiles.

**Figure 4:**
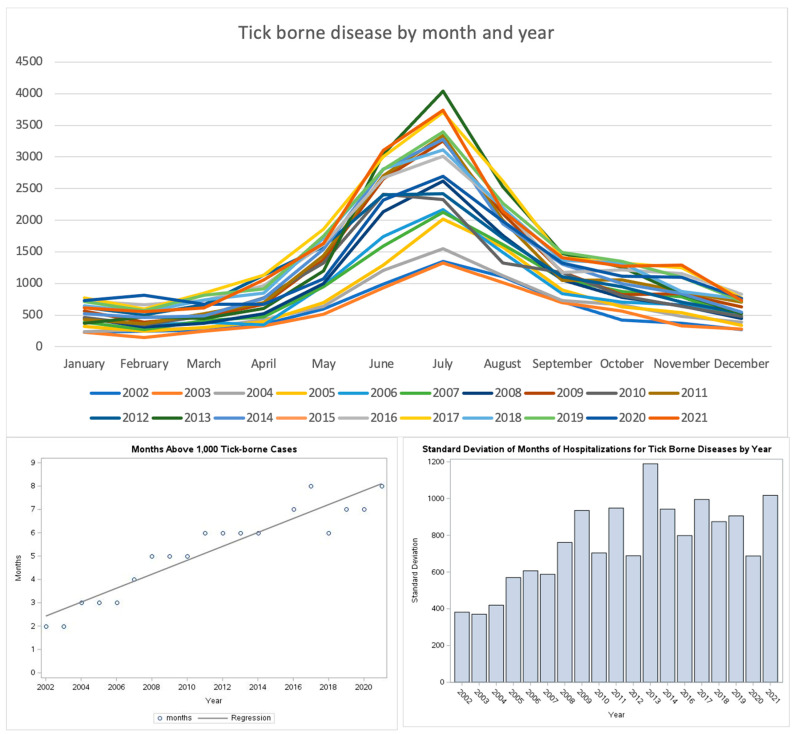
Change in the length of seasons for tick-borne disease hospitalizations.

Seasonality: Analysis of seasonality and length showed that hospitalizations for TBDs occurred year-round since the beginning of the study period; there were consistent peaks for all TBDs in the summer, with July having the highest frequency for almost every year (Figure 4). The lengthening of the season was addressed in two ways. The standard deviation of the peak frequency in number of months for each year was plotted, showing a general upward trend. In a second analysis, the number of months in which there were at least 1000 hospital admissions were plotted. A line of best fit was plotted, showing a significant rise in the number of months in which 1000 hospitalizations for TBDs occurred. In 2000, approximately 2.5 months of the year with at least 1000 hospitalizations to almost 8 months in the year in 2021.

## 4. Discussion

The findings of our study highlight the overall increase in hospitalizations related to TBDs in the United States over the study period from 2002 to 2021. Lyme disease accounted for most hospitalizations. However, interesting findings were noted for the other TBDs, including ehrlichiosis, anaplasmosis, babesiosis, and Rocky Mountain Spotted Fever (RMSF), with each demonstrating regional clustering, changes during the seasons, and various spikes overtime. Similar patterns of spread for TBDs have been found in other studies [11,12].

Geographic analysis showed that the burden of the disease increases was shown in the Northeast and Upper Midwest regions. The *Ixodes scapularis* tick is endemic to these regions, and with the changes in land use and climate over the study period, there is reasonable suspicion to believe that those factors have contributed to the amplification of tick activity and populations. The Pacific region did not show drastic changes, and any increase could be due to variations in endemic species of vectors and other host ecology. It is important to note that the resurgence of the white-tailed deer population, specifically in the Northeast and Upper Midwest, may have played a role in contributing to the expanding tick populations and, therefore, the increased hospitalization burden of TBDs in these regions [9,13].

The highest numbers of TBD hospitalizations occur in June and July. TBD hospitalizations are increasing year-round, with the fastest rate of increase in the summer months. Our study cannot attribute a cause to this, but multiple factors may play a role. These include demographic changes such as population increase. Other factors may include expanding endemic range and the increased population of host species. The present study shows a strong association between the increasing length of the season of hospitalizations for tick-borne diseases and time over the past 20 years. The changes in climate factors are known to not only favor expansion of geographic range, population, and season of the tick vectors, but also increase the factors impacting tick activity, including increased feeding and the length of time of attachment. Richard Ostfeld and Jesse Brunner also suggested that increased tick activity is due to climate change through their mechanistic and phenomenological models, suggesting the need for more studies on the influence of climate in tick-borne pathogen dynamics [14].

Demographic analysis showed that most hospital cases were among older adults, with the majority of patients being white, male, and privately insured individuals. This could simply be a representation of overall population demographics but raises the question of the effect of healthcare access on hospitalization for tick-borne disease. It remains unclear whether these groups are more likely to receive inpatient care when infected.

There is an overwhelmingly Caucasian predominance among TBD hospitalizations at 88% of hospitalizations, followed distantly by Hispanic and Black patients at about 4% each. All TBDs have a strong Caucasian predominance—between 88 and 91%—for Rickettsial hospitalizations, which are 66% Caucasian. About 20% of rickettsial cases are seen in Hispanic populations in our data (Table 3). NIS race determination is based on self-identification and uses only White, Black, Hispanic, Asian/Pacific Islander, Native American, and Other. This is different from the US census bureau, which is more complicated and detailed, where Hispanic falls into an ethnic category rather than a racial category. The census data includes people who identify as White alone, white Hispanic, and non-white Hispanic, making it difficult to compare. However, in the 2020 census, Hispanic or Latino populations represented 19.5% of the US population, identical to the rickettsial diseases group. Male patients, in general, are shown to present with TBDs more frequently than female patients. The higher incidence of TBDs in white Caucasians has been attributed to their higher indulgence of outdoor activities like hiking, camping, gardening, etc., which could also explain their higher incidence in males [15]. Studies of rickettsial diseases like Rocky Mountain Spotted Fever have revealed a higher incidence among White males; however, a high incidence of these diseases has not been seen among Hispanic populations as in our data. Rather, many studies have shown that the rate of rickettsial infections is increasing in Native Americans [16,17]. Despite TBDs being more commonly seen in Caucasians, there are worse outcomes in Black patients and other minorities due to difficulty in seeing rashes like erythema migrans or failure to seek early medical care [18].

Our study also highlights the growing burden of hospitalization for TBDs in the United States. The median cost per patient has increased from USD 9333 in 2002 to USD 35,161 in 2021, amounting to a weighted total cost of more than USD 1 billion. This highlights the growing need for prevention as well as early diagnosis and treatment of TBDs.

Routine testing for coinfection in patients with Lyme disease is not currently recommended by US guidelines [19]. However, coinfection testing is currently recommended when a patient with Lyme disease has uncharacteristic symptoms indicating risk of coinfection or persistent fever after starting the appropriate antibiotics. This study revealed a high proportion of coinfection between Lyme, babesiosis, and ehrlichiosis/anaplasmosis among patients hospitalized with TBDs. Notably, babesiosis requires a different treatment regimen than Lyme or ehrlichiosis/anaplasmosis. These results suggest that further study into the utility of routine coinfection testing for hospitalized patients with one of these TBDs should be carried out.

Importantly, the study addresses a gap in the literature. Passive surveillance systems such as the CDC’s National Notifiable Diseases Surveillance System are known to underestimate case counts [5]. Our use of the inpatient data offers an additional perspective that helps further characterize the burden of TBDs, especially for severe cases where hospitalization is required.

The limitations of this study are related to the nature of the HCUP database. The database only includes inpatient data, and many TBDs are treated in an outpatient setting. Disease identification relies on ICD codes. These codes can be entered inaccurately by treating physicians and hospital coders. The study time period includes the transition between ICD9 and ICD10, and it is challenging to match diagnoses across these coding systems. For example, there is not a code for anaplasmosis in ICD9, while there is one in ICD10. The change to ICD10 occurred in 2015, which caused the data from that year to be incomplete and unusable in our analysis. Each hospitalization is counted as a separate entry in the HCUP database; thus, the same patient could be coded twice if admitted more than once for the same condition.

Ultimately, the findings of this study show increasing morbidity associated with TBDs, necessitating ongoing research into prevention, accurate diagnosis, and therapeutics. Furthermore, public health messaging should evolve, given regional and seasonal changes in TBD hospitalizations. Clinician awareness of these findings is key to maintaining vigilance for these increasingly common conditions.

## 5. Conclusions and Future Directions

This study highlights the rising burden of TBD hospitalizations across the United States. As ecosystems around the country adapt to climate changes every year, the public health sector needs to be aware of these changes. Lyme disease is still a predominant TBD that requires hospitalization, but it is not the only TBD. Hospitalizations from other TBDs like babesiosis, ehrlichiosis, and anaplasmosis warrant attention. Coinfection is also common among TBD hospitalizations. Seasonal and regional distribution of all TBDs in the United States is expanding.

This lays the foundation for future research into TBD prevention, diagnosis, and treatment. Changing geographic and seasonal patterns necessitate adjustments to surveillance and prevention strategies. This study helps characterize the inpatient burden of TBDs; however, similar reviews of the burden in an outpatient setting are warranted. Racial disparities in TBD hospitalization require further characterization with an emphasis on understanding whether there are social determinants of health preventing minority patients from seeking medical care for TBDs. The high incidence of coinfection suggests investigation into routine coinfection testing or cascading testing algorithms.

## Figures and Tables

**Table 1:** Inclusion criteria billing codes for tick-borne diseases (TBDs).

Disease	ICD9	2002–2014	ICD 10	2016–2021
Lyme disease	08881	Lyme disease	A6920	Lyme disease, unspecified
			A6921	Meningitis due to Lyme disease
			A6922	Other neurologic disorders in Lyme disease
			A6923	Arthritis due to Lyme disease
			A6929	Other conditions associated with Lyme disease
Babesiosis	08882	Babesiosis	B600	Babesiosis
			B6000	Babesiosis, unspecified
			B6001	Babesiosis due to *Babesia microti*
			B6002	Babesiosis due to *Babesia duncani*
			B6003	Babesiosis due to *Babesia divergens*
			B6009	Other babesiosis
Ehrlichiosis and anaplasmosis	08240	Ehrlichiosis, unspecified	A7740	Ehrlichiosis, unspecified
	08241	Ehrlichiosis chafeensis [*E. chafeensis*]	A7741	*Ehrlichiosis chafeensis* [*E. chafeensis*]
	08249	Other ehrlichiosis	A7749	Other ehrlichiosis
			A7981	Rickettsiosis due to *Ehrlichia sennetsu*
			A7982	Anaplasmosis [*A. phagocytophilum*]
Tularemia	0210	Ulceroglandular tularemia	A210	Ulceroglandular tularemia
	0211	Enteric tularemia	A211	Oculoglandular tularemia
	0212	Pulmonary tularemia	A212	Pulmonary tularemia
	0213	Oculoglandular tularemia	A213	Gastrointestinal tularemia
	0218	Other specified tularemia	A217	Generalized tularemia
	0219	Unspecified tularemia	A218	Other forms of tularemia
			A219	Tularemia, unspecified
Rickettsia fevers	0832	Rickettsialpox	A770	Spotted fever due to *Rickettsia rickettsii*
	3231	Encephalitis, myelitis, encephalomyelitis in rickettsial diseases	A771	Spotted fever due to *Rickettsia conorii*
	0829	Tick-borne rickettsiosis, unspecified	A772	Spotted fever due to *Rickettsia siberica*
	0839	Rickettsiosis, unspecified	A773	Spotted fever due to *Rickettsia australis*
	0828	Other specified tick-borne rickettsioses	A778	Other spotted fevers
	0829	Tick-borne rickettsiosis, unspecified	A779	Spotted fever, unspecified
			A752	Typhus fever due to *Rickettsia typhi*
			A753	Typhus fever due to *Rickettsia tsutsugamushi*
			A770	Spotted fever due to *Rickettsia rickettsii*
			A771	Spotted fever due to *Rickettsia conorii*
			A772	Spotted fever due to *Rickettsia siberica*
			A773	Spotted fever due to *Rickettsia australis*
			A791	Rickettsialpox due to *Rickettsia akari*
			A799	Rickettsiosis, unspecified
Other TBD				
Tick-borne relapsing fever	0871	Relapsing fever, tick-borne	A681	Tick-borne relapsing fever
Powassan virus disease			A8481	Powassan virus disease
Tick-borne encephalitis	0638	Other specified tick-borne viral encephalitis	A840	Far Eastern tick-borne encephalitis
	0639	Tick-borne viral encephalitis, unspecified	A841	Central European tick-borne encephalitis
			A848	Other tick-borne viral encephalitis
			A8489	Other tick-borne viral encephalitis
			A849	Tick-borne viral encephalitis, unspecified
Colorado Tick Fever	0661	Tick-borne fever	A932	Colorado tick fever
Tick-borne hemorrhagic fevers	0650	Crimean hemorrhagic fever [CHF Congo virus]	A980	Crimean-Congo hemorrhagic fever
	0651	Omsk hemorrhagic fever	A981	Omsk hemorrhagic fever
	0653	Other tick-borne hemorrhagic fever		
Kyasanur Forest disease	0652	Kyasanur forest disease	A982	Kyasanur Forest disease

**Table 2:** Baseline demographic data.

Numeric Variables	Mean (Std-Error)	Median (IQR)	Q1–Q3
**Age (years)**	53.43 (0.22)	56.02 (31.50)	38.32–69.82
**Length of Stay (days)**	4.96 (0.04)	2.87 (3.56)	1.58–5.14
**Hospitalization Charge (USD)**	39,564 (585)	20,225 (29,784)	10,728–40,512

**Table 3:** Detailed demographic data for tick-borne diseases (TBDs).

Characteristic		All TBDs	Lyme	Babesiosis	Ehrlichiosis Anaplasmosis	Tularemia	Rickettsia	Other TBD
Total Discharges		261,630	171,328	18,057	46,446	2428	16,412	19,435
Sex	Male	141,110 (53.9%)	85,270 (49.8%)	11,701 (64.8%)	27,809 (59.9%)	1610 (66.3%)	9775 (59.6%)	12,460 (64.1%)
	Female	120,463 (46.0%)	86,020 (50.2%)	6355 (35.2%)	18,637 (40.1%)	818 (33.7%)	6626 (40.4%)	6965 (35.8%)
In-hospital mortality		2678 (1.02%)	1520 (0.89%)	372 (2.06%)	535 (1.15%)	*	188 (1.15%)	133 (0.68%)
Race		Total (%)						
Caucasian		212,894 (87.28%)	142,895 (88.68%)	13,830 (80.22%)	38,193 (91.11%)	1858 (86.34%)	10,487 (67.74%)	15,939 (90.25%)
Black		9231 (3.78%)	5854 (3.63%)	693 (4.02%)	1261 (3.01%)	71 (3.30%)	934 (6.03%)	717 (4.06%)
Hispanic		11,662 (4.78%)	6152 (3.82%)	1291 (7.49%)	1060 (2.53%)	132 (6.13%)	3021 (19.51%)	507 (2.87%)
Asian and Pacific Islander		2850 (1.17%)	1670 (1.04%)	567 (3.29%)	437 (1.04%)	*	253 (1.63%)	126 (0.71%)
Native American		908 (0.37%)	378 (0.23%)	39 (0.23%)	186 (0.44%)	*	206 (1.33%)	108 (0.61%)
Other		6371 (2.61%)	4189 (2.60%)	820 (4.76%)	782 (1.87%)	91 (4.23%)	580 (3.75%)	263 (1.49%)
Total Race		243,916	161,138	17,240	41,919	2152	15,481	17,660
Median income		Total (%)						
0–25th percentile		41,321 (16.58%)	18,934 (11.64%)	1010 (5.84%)	8200 (18.42%)	839 (37.19%)	6968 (44.25%)	6887 (36.93%)
26th to 50th percentile (median)		51,423 (20.64%)	31,737 (19.51%)	2107 (12.19%)	10,971 (24.65%)	524 (23.23%)	3569 (22.66%)	4520 (24.24%)
51st to 75th percentile		60,972 (24.47%)	41,772 (25.68%)	4523 (26.17%)	10,773 (24.20%)	524 (23.23%)	2885 (18.32%)	3475 (18.63%)
76th to 100th percentile		95,431 (38.30%)	70,236 (43.17%)	9641 (55.79%)	14,571 (32.73%)	369 (16.36%)	2326 (14.77%)	3766 (20.20%)
Total Median Income		249,147	162,679	17,281	44,515	2256	15,748	18,648
Payer		Total (%)						
Medicare		99,167 (38.00%)	60,409 (35.34%)	9515 (52.92%)	22,977 (49.64%)	599 (25.01%)	4297 (26.25%)	7278 (37.53%)
Medicaid		26,692 (10.23%)	18,624 (10.89%)	1369 (7.61%)	2914 (6.30%)	370 (15.45%)	2772 (16.93%)	1470 (7.58%)
Private		115,574 (44.28%)	80,922 (47.34%)	5923 (32.94%)	16,982 (36.69%)	1118 (46.68%)	7141 (43.62%)	8419 (43.42%)
Self-pay		11,547 (4.42%)	6223 (3.64%)	689 (3.83%)	1891 (4.09%)	174 (7.27%)	1642 (10.03%)	1375 (7.09%)
No Charge		880 (0.34%)	482 (0.28%)	72 (0.40%)	115 (0.25%)	*	105 (0.64%)	104 (0.54%)
Other		7121 (2.73%)	4290 (2.51%)	412 (2.29%)	1411 (3.05%)	134 (5.59%)	414 (2.53%)	745 (3.84%)
Total Payer		260,981	170,950	17,980	46,290	2395	16,371	19,391
Region		Total (%)						
Northeast		138,505 (52.94%)	103,046 (60.15%)	15,691 (86.90%)	22,588 (48.63%)	310 (12.76%)	1716 (10.46%)	3322 (17.09%)
Midwest		41,691 (15.94%)	22,468 (13.11%)	1168 (6.47%)	12,442 (26.79%)	824 (33.92%)	2290 (13.95%)	4256 (21.90%)
South		69,010 (26.38%)	35,985 (21.00%)	846 (4.69%)	11,112 (23.92%)	961 (39.56%)	11,101 (67.64%)	11,236 (57.81%)
West		12,424 (4.75%)	9828 (5.74%)	352 (1.95%)	304 (0.65%)	334 (13.75%)	1304 (7.95%)	621 (3.20%)
Total Region		261,620	171,327	18,057	46,446	2429	16,411	19,435
Medical center type		Total (%)						
Rural		18,496 (16.85%)	9096 (12.44%)	663 (10.57%)	4819 (25.96%)	304 (28.63%)	975 (22.76%)	3541 (32.05%)
Urban		91,266 (83.15%)	64,032 (87.56%)	5609 (89.43%)	13,741 (74.04%)	758 (71.37%)	3309 (77.24%)	7507 (67.95%)
Total Medical Center Type		109,762	73,128	6272	18,560	1062	4284	11,048

* Per NIS requirements, cells containing fewer than ten patients cannot be reported to the researchers or published due to the potential risk of re-identifying patients.

**Table 4:** TBD frequencies by lowest income quartile: highest income quartile.

Total	Lyme	Babesiosis	Ehrlichiosis/Anaplasmosis	Tularemia	Rickettsia	Other TBD
0.43	0.27	0.10	0.56	2.27	3.00	1.83

**Table 5:** Charges for weighted TBD hospital discharges.

Year	Median Charge Per Patient	Total US of Weighted Hospitalization Charges
2002	USD 9433	USD 114,053,728
2003	USD 10,308	USD 130,393,822
2004	USD 11,383	USD 159,911,284
2005	USD 11,491	USD 195,805,392
2006	USD 12,515	USD 223,942,432
2007	USD 14,693	USD 272,277,372
2008	USD 14,582	USD 310,389,585
2009	USD 15,819	USD 428,942,037
2010	USD 15,373	USD 375,369,440
2011	USD 17,514	USD 470,149,290
2012	USD 18,268	USD 460,113,240
2013	USD 19,693	USD 582,070,125
2014	USD 20,589	USD 578,244,050
2016	USD 24,481	USD 813,124,410
2017	USD 26,356	USD 933,416,825
2018	USD 28,303	USD 884,497,920
2019	USD 31,856	USD 1,108,943,360
2020	USD 35,161	USD 1,043,491,160
2021	USD 38,985	USD 1,265,175,720

**Table 6:** Co-infection weight frequencies.

Concurrent TBD Infections	Weighted Frequency
Lyme + Babesiosis	4751
Lyme + Ehrlichiosis/Anaplasmosis	3915
Lyme + Tularemia	*
Lyme + Rickettsia	379
Lyme + Other TBD	442
Babesiosis + Ehrlichiosis/Anaplasmosis	1510
Babesiosis + Tularemia	*
Babesiosis + Rickettsia	54
Babesiosis + Other TBD	144
Ehrlichiosis/Anaplasmosis + Tularemia	59
Ehrlichiosis/Anaplasmosis + Rickettsia	596
Ehrlichiosis/Anaplasmosis + Other	1181
Tularemia + Rickettsia	*
Tularemia + Other TBD	80
Rickettsia + Other TBD	105

* Per NIS requirements, cells containing fewer than ten patients cannot be reported to the researchers or published due to the potential risk of re-identifying patients.

**Table 7:** Number and percentage of TBD coinfections.

Concurrent TBD Infections	Total	Total Coinfections	Percentage of Coinfection
Lyme	171,328	9487	5.5
Babesiosis	18,057	6459	35.8
Ehrlichiosis/Anaplasmosis	46,446	7261	15.6
Tularemia	2428	138	5.7
Rickettsia	16,412	1135	6.9

## Data Availability

The data presented in this study are openly available in HCUP National Inpatient Sample (NIS). Healthcare Cost and Utilization Project (HCUP). 2012. Agency for Healthcare Research and Quality, Rockville, MD.
